# Characterization of the first naturally thermostable terpene synthases and development of strategies to improve thermostability in this family of enzymes

**DOI:** 10.1111/febs.14072

**Published:** 2017-05-03

**Authors:** Matthew Q. Styles, Edward A. Nesbitt, Scott Marr, Marc Hutchby, David J. Leak

**Affiliations:** ^1^ Department of Biology and Biochemistry University of Bath UK; ^2^ Department of Chemistry University of Bath UK

**Keywords:** kinetics, structural model, terpenoid, thermophile, thermostability

## Abstract

The terpenoid family of natural products is being targeted for heterologous microbial production as a cheaper and more reliable alternative to extraction from plants. The key enzyme responsible for diversification of terpene structure is the class‐I terpene synthase (TS), and these often require engineering to improve properties such as thermostability, robustness and catalytic activity before they are suitable for industrial use. Improving thermostability typically relies on screening a large number of mutants, as there are no naturally thermostable TSs described upon which to base rational design decisions. We have characterized the first examples of natural TSs exhibiting thermostability, which catalyse the formation of the sesquiterpene τ‐muurolol at temperatures up to 78 °C. We also report an enzyme with a *k*_cat_ value of 0.95 s^−1^ at 65 °C, the highest *k*_cat_ recorded for a bacterial sesquiterpene synthase. In turn, these thermostable enzymes were used as a model to inform the rational engineering of another TS, with the same specificity but low sequence identity to the model. The newly engineered variant displayed increased thermostability and turnover. Given the high structural homology of the class‐I TS domain, this approach could be generally applicable to improving the properties of other enzymes in this class.

**Database:**

Model data are available in the PMDB database under the accession number PM0080780.

## Graphical abstract

This study has characterized the first naturally thermostable class‐I sesquiterpene synthases; enzymes that catalyse the formation of the sesquiterpene τ‐muurolol and can retain activity as high as 78 °C, and have the highest *k*_cat_ value (0.95 s^−1^ at 65 °C) observed for a bacterial terpene synthase to date.

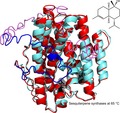

**Abbreviations**
CAPS: *N*‐cyclohexyl‐3‐aminopropanesulfonic acid
DCM: dichloromethane
DSC: differential scanning calorimetry
FID: flame ionization detector
FPP: farnesyl pyrophosphate
IPTG: isopropyl β‐D‐1‐thiogalactopyranoside
LB: Luria–Bertani medium
MES: 2‐(*N*‐morpholino)ethanesulfonic acid
MWCO: molecular weight cut‐off
OD600: optical density at a wavelength of 600 nm
PIPES: piperazine‐*N*,*N*′‐bis(2‐ethanesulfonic acid)
RMSD: root‐mean‐square deviation
TBA‐FPP: tetrabutylammonium farnesyl pyrophosphate
*T*_m_: melt temperature
Tris/HCl: tris(hydroxymethyl)aminomethane hydrochloride salt
TS: terpene synthase

## Introduction

Enzyme stability is often the limiting factor in the development of commercial enzymes 1, 2. In particular, thermal stability is essential in a wide range of industrial applications, including the household care, food, textiles and bioenergy industries, where enzymes are required to be robust under a range of temperature conditions. Additionally, thermostable enzymes are desirable as starting points for directed evolution of new functions, as subsequent mutants can better tolerate changes that would otherwise have led to destabilization 3. Class‐I terpene synthases (TSs) typically have low activity and stability, and so are attractive targets for the engineering of increased thermostability and thermoactivity 4. However, no naturally thermostable examples have been described, and so there is no model for thermostability in this class of enzymes.

Class‐I TSs produce terpenoids, the single largest class of natural products, with over 70 000 members in the natural products database, including examples in pharmaceuticals, fragrances, flavours, polymers and biofuels 5, 6, 7, 8, 9. The extraordinary diversity of terpenoids is partly driven by class‐I TSs, which convert C_10_, C_15_ and C_20_ isoprenyl pyrophosphates into monoterpenes, sesquiterpenes and diterpenes respectively. For example, the C_15_ sesquiterpene synthases use farnesyl pyrophosphate (FPP) as a substrate to generate over 300 carbon skeletons 10, 11. In turn, these sesquiterpenes can be processed further either enzymatically or chemically to generate the huge range of sesquiterpenoid natural products 12, 13. Traditionally, valuable terpenes have always been extracted directly from the plants that natively produced them, but in the last decade, there have been substantial efforts put into obtaining high‐value sesquiterpenes via microbial fermentation. Such microbial platforms require TSs that are expressed and active at fermentation temperatures. Currently, most known sesquiterpene synthases are found in plants 14, 15, with increasing numbers more recently discovered in bacteria 16; to this date none of these have been characterized as thermostable, and many are optimally expressed at temperatures below 30 °C 13, 17.

In order to make TSs useful industrially, protein engineering is required to improve enzyme properties, including thermostability, without impacting product specificity. There have been no examples of naturally thermostable class‐I TSs described but recently there have been several efforts to create thermostable variants using generically applicable techniques 18, 19. Diaz *et al*. were able to generate a thermostable variant of tobacco *epi*‐aristolochene synthase using computational methods, with activity observed up to 65 °C. However, the thermostable mutant was expressed in insoluble form and had lost much of its activity and specificity compared to the wild‐type 18. Another approach developed a presilphiperfolan‐8β‐ol synthase towards thermostability using a directed evolution approach, leading to an enzyme that retained full activity up to 50 °C (with a *T*_50_ value of 54 °C) 19. Both approaches for generating thermostable TSs could be informed by model examples from nature that retain activity up to high temperatures.

The class‐I TS active site is contained within an α‐helical domain, containing two conserved Mg^2+^‐binding motifs coordinating three Mg^2+^ ions. In turn, the Mg^2+^ ions coordinate the diphosphate head group of the substrate, while the isoprenyl tail positions within the binding pocket 20. A cascade of intramolecular reactions, often including numerous cyclizations and rearrangements, can result in remarkable specificity for a single product 21. However, most TSs have promiscuous activity and will produce a range of products, and preference for a particular product can be tipped by relatively few active‐site mutations 21, 22. While those TSs for which a structure is known share a high structural homology, outside the conserved motifs there is generally low sequence identity, especially in bacterial examples 16, 23. There is a possibility therefore that some of the structural features that affect thermostability and those that affect product specificity can be considered separately, and that by using a thermostable TSs as a model, increased thermostability can be engineered into a variant from a mesophile.

In this work, two enzymes were characterized as the first examples of thermostable sesquiterpene synthases. The enzymes RoseRS_3509 and Rcas_0622 had previously been functionally identified *in vivo* as τ‐muurolol synthases by Yamada *et. al*. 16. We demonstrate that one of the enzymes, RoseRS_3509, has a higher turnover rate than any other bacterial TS, and that these two enzymes can be used as the first model for thermostability in this class of enzymes.

## Results

### Identification of thermostable terpene synthases

Potential thermostable TSs were identified using the HMMER web server, which uses profile hidden Markov models to return potential sequences of interest 24. In this case, the sequence for the bacterial τ‐muurolol synthase, SSCG_03688 (Accession Number: EDY50541.1), was used as an initial entry, and the output genes were searched for thermophilic native organisms. Gene sequences were codon optimized and expressed in *Escherichia coli*, then screened for TS activity. Two TSs were identified, RoseRS_3509 from *Roseiflexus* sp.RS‐1 (AN: WP_011958209.1) and Rcas_0622 from *Roseiflexus castenholzii* DSM 13941. The two *Roseiflexus* protein sequences, recently identified as τ‐muurolol synthases using a similar search strategy 16, shared just 32% identity with SSCG_03688 with 94% and 89% coverage respectively. For context, a blast of the *Rosieflexus* protein sequences search reveals over 1000 putative bacterial TS proteins with similar identity (27–35% identity, > 90% coverage). Production of τ‐muurolol as the major product was confirmed by GC‐MS.

### Preliminary screening using tris(tetrabutylammonium) farnesyl pyrophosphate

In previous reports describing *in vitro* TS activity, the ammonium salt of FPP has been used 17, 21, 25, which is complicated to prepare and expensive to buy. We discovered that the tetrabutylammonium salt of FPP (TBA‐FPP), which could be prepared readily and economically from farnesol, was also an effective substrate for each of the sesquiterpene synthases we tested. For preliminary screening of TSs, it was feasible to use TBA‐FPP assays in place of the ammonium salt, allowing the determination of suitable assay parameters including assay time, enzyme concentration and temperature range for each enzyme. However, in order to provide consistency with previous literature, all experimental data reported in this manuscript used the commercial ammonium salt of FPP as substrate.

### Characterization of terpene synthase thermoactivity

**Figure 1:**
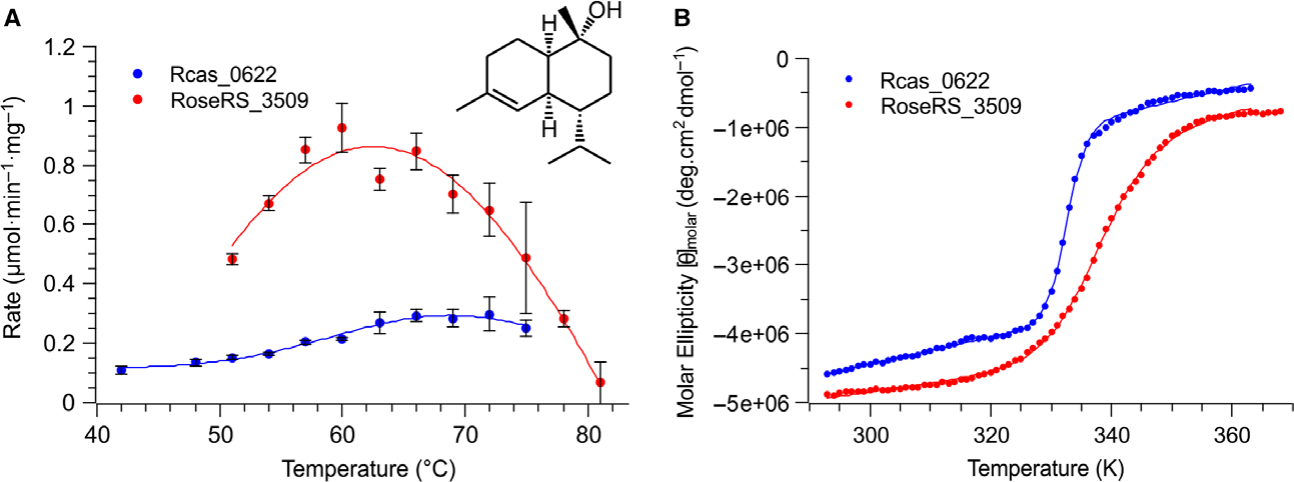
(A) Thermoactivity profile of the two thermostable sesquiterpene synthases, RoseRS_3509 and Rcas_0622. The error bars represent the standard error of mean for three replicates. Inset; the structure of the sesquiterpene product, τ‐muurolol. (B) CD absorption at 222 nm for the two thermostable sesquiterpene synthases. The lines show the curves fit in order to determine the *T*_m_ of the proteins.

Both enzymes were found to be sesquiterpene synthases, converting FPP into τ‐muurolol as the major product *in vitro*. The thermoactivity of each enzyme was assessed over 1 min and the optimum temperature for activity was determined to be 61 °C for RoseRS_3509 and 69 °C for Rcas_0622 (Fig. 1A). For RoseRS_3509, activity was still detected at 78 °C, which to our knowledge represents the highest temperature for which activity in sesquiterpene synthases has ever been reported. Moreover, given the notoriously loose product profile of such enzymes, it is significant that the product profile remained unchanged at higher temperatures, as determined by both GC‐MS and the flame ionization detector (FID) peak area.

### Characterization of terpene synthase thermostability

The melting profile for the two enzymes were determined by monitoring the characteristic α‐helix circular dichroism (CD) signal at 222 nm, from 20 to 94 °C, as a surrogate for enzyme thermostability. The CD signal at 222 nm was fitted using a simple two‐state transition equation, adapted from Catici *et al*. 26, with *R* ^2^ > 0.99 in both cases. Fitting the data gave a melt temperature (*T*_m_) of 64.8 ± 0.1 °C for RoseRS_3509 and 59.6 ± 0.1 °C for Rcas_0622 (Fig. 1B), where the error represents the deviation from the fit.

### Kinetics of thermostable terpene synthases

Both thermostable TSs were found to follow single‐substrate Michaelis–Menten kinetics at 65 °C. Kinetic parameters were measured at 65 °C by carrying out 1‐min incubations over a range of FPP concentrations from 1.2 to 92 μm giving the kinetic values shown in Table 1. The product was quantified using GC‐FID, and the *k*_cat_ value of 0.95 s^−1^ for RoseRS_03509 is the highest *k*_cat_ value reported for a bacterial sesquiterpene synthase at any temperature.

**Table 1:** Kinetic parameters of thermostable TSs at 65 °C

Enzyme	*k*_cat_ (s^−1^)	*K*_M_ (μm)
RoseRS_3509	0.95 ± 0.29	87 ± 31
Rcas_0622	0.090 ± 0.008	5.9 ± 2.0

### Sequence alignment and modelling

**Figure 2:**
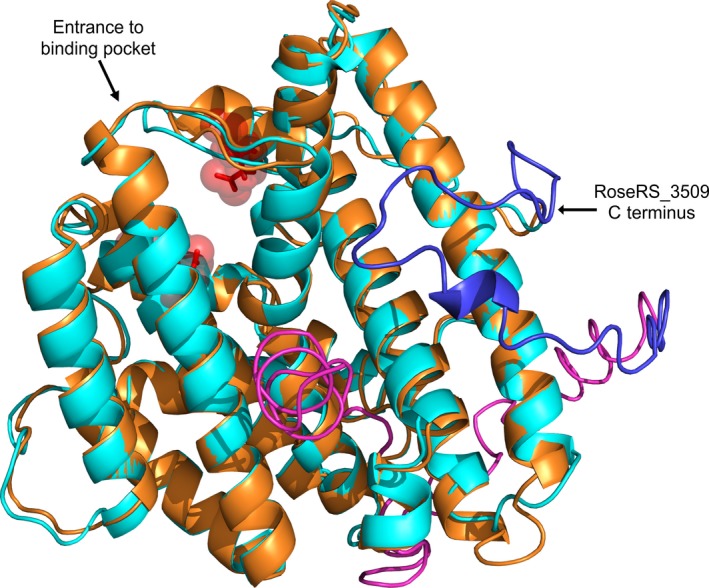
Overlaid models for RoseRS_3509 (orange, estimated RMSD = 3.7 ± 2.5 Å) and SSCG_03688 (cyan, estimated RMSD = 5.0 ± 3.2 Å) demonstrate the predicted structural homology between the two enzymes, despite the low sequence identity. The mesophile enzyme has an extended C terminus compared to the thermostable variants; the SSCG_03688 R6 mutant tail is shown in blue, and the full length wild‐type tail is shown in magenta. Two of the Mg^2+^ binding residues are at the entry to the active site and are shown in red.

The amino acid sequences of the two thermostable TSs investigated, RoseRS_3509 and Rcas_0622, were aligned with the mesophile τ‐muurolol synthase, SSCG_03688. This showed that overall there was good similarity between the three throughout most of the sequence, with two notable exceptions. Firstly, the thermostable variants are significantly truncated at the C terminus relative to SSCG_03688, where the C terminus extends about 100 residues further. As a result, SSCG_03688 is 418 amino acids in length, while RoseRS_3509 has just 326 amino acids. Secondly, the region directly following the first magnesium‐binding site motif (amino acids 89–118 in SSCG_03688) has low similarity with the two thermostable sequences. In order to observe the relationship of the low homology regions to the overall enzyme structure, both sequences were modelled using the I‐TASSER server 27, 28 and the models were overlaid using pymol (Fig. 2). The model for RoseRS_3509 has an estimated root‐mean‐square deviation (RMSD) = 3.7 ± 2.5 Å, while the model for SSCG_03688 has RMSD = 5.0 ± 3.2 Å. It is clear that the first region of low similarity occurs in a loop immediately following one of the magnesium‐binding sites, and is unstructured in SSCG_03688, but appears to have more α‐helical structure in RoseRS_3509. The C‐terminal region in SSCG_03688 that is not present in RoseRS_3509 appears to wrap 180˚ around the barrel of the protein, and has some β‐sheet structure (Fig. 2).

### Truncation of SSCG_03688

**Figure 3:**
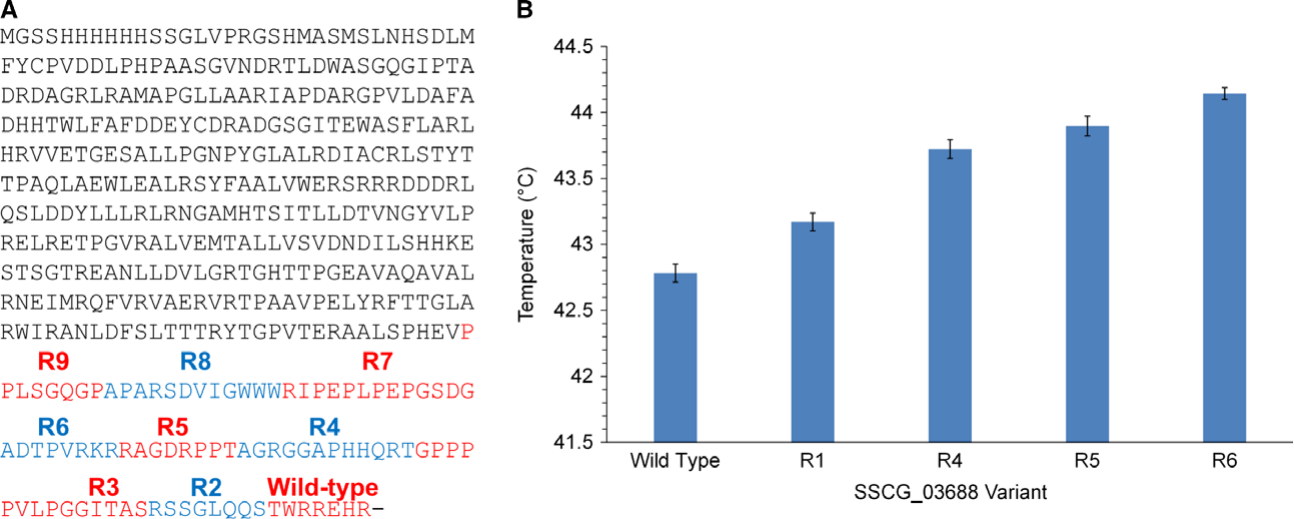
(A) The sequence of the SSCG_03688 protein, with the termini of the truncated mutants created in this study marked. (B) The *T*_m_ of the wild‐type SSCG_03688 and truncated mutants, as derived from their respective CD melt profiles at 222 nm. The error bars represent the deviation of the *T*_m_ variable from the fit described in Eqn (1).

In the directed evolution study by Lauchli *et al*. 19, it was found that the most thermostable TS generated had an early stop codon, resulting in a three‐amino‐acid truncation of the C terminus. Given that the thermostable examples also had shortened C‐terminal regions than SSCG_03688, the effect of C‐terminal truncation on thermostability was investigated. A set of mutants of SSCG_03688 was created in which the C terminus was sequentially shortened by removing blocks of 7–15 amino acids (R2–R9), with the final mutant having a C terminus of a comparable length to RoseRS_3509 (Fig. 3A). The mutants were expressed, purified, and melting curves were determined by CD. To improve the accuracy of the comparison, melting temperatures were determined by fitting the data using Eqn (1) in the same way as described for the thermostable TSs, where each curve was fit with *R* ^2^ > 0.99. The CD data show that removing blocks of amino acids from the C terminus does indeed lead to a sequential increase in melting temperature, with the *T*_m_ increasing by up to 1.4 °C with the R6 mutant (Fig. 3B). However, further truncation of the C terminus beyond R6 (i.e. R7–R9) affected protein folding in *E. coli*, resulting in heterologous expression of insoluble protein aggregates.

### Differential scanning calorimetry (DSC)

**Figure 4:**
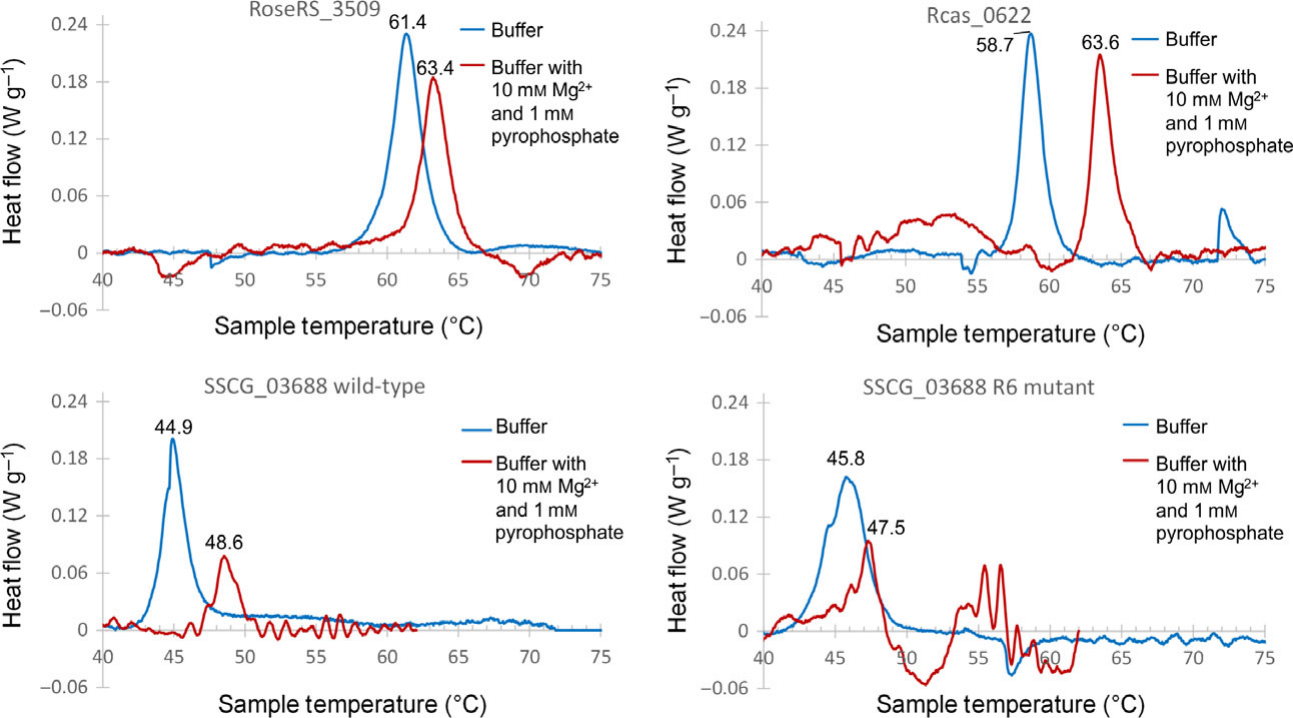
DSC traces for each protein. Red traces were recorded in buffer containing Mg^2+^ and pyrophosphate. Blue traces were recorded in the absence of Mg^2+^ and pyrophosphate. The *T*_m_ for each trace is shown the legend.

The *T*_m_ was also determined for the TSs investigated using differential scanning calorimetry (DSC) in native activity buffers. In particular, the *T*_m_ was determined in the presence and absence of Mg^2+^ and pyrophosphate, which have been shown to induce the closed and open state respectively of other TSs 29. Changing the buffer to include both Mg^2+^ and pyrophosphate resulted in a small but significant increase in *T*_m_ for RoseRS_3509 and Rcas_0622, by 2.0 and 4.9 °C respectively, with no change in peak shape (Fig. 4). DSC carried out on SSCG_03688 and the R6 mutant demonstrated the same trend of *T*_m_ increase for the mutant as determined by CD, with *T*_m_ increasing from 44.9 to 45.8 °C. The introduction of Mg^2+^ and pyrophosphate again appeared to result in a *T*_m_ shift to higher temperatures for both SSCG_03688 variants, and also led to a substantially altered peak shape and more convoluted baseline, making firm conclusions impossible for the R6 mutant (Fig. 4).

### Kinetics of SSCG_03688 R6 truncated mutant

Kinetic parameters were determined at 30 °C for both the wild‐type enzyme and the most truncated mutant expressed (R6, Table 2). The *k*_cat_ value reported here for the wild‐type is higher than previously reported 17, but consistent with the findings that GC‐FID vial assays give relatively higher values than radioactivity assays for TSs 30.

**Table 2:** Kinetic parameters of SSCG_03688 and truncated mutant

Enzyme	*k*_cat_ (s^−1^)	*K*_M_ (μm)
SSCG_03688 wild‐type	0.018 ± 0.001	4.5 ± 1.0
SSCG_03688 R6	0.083 ± 0.006	16.3 ± 4.0

### Thermoactivity of SSCG_03688 R6 truncated mutant

**Figure 5:**
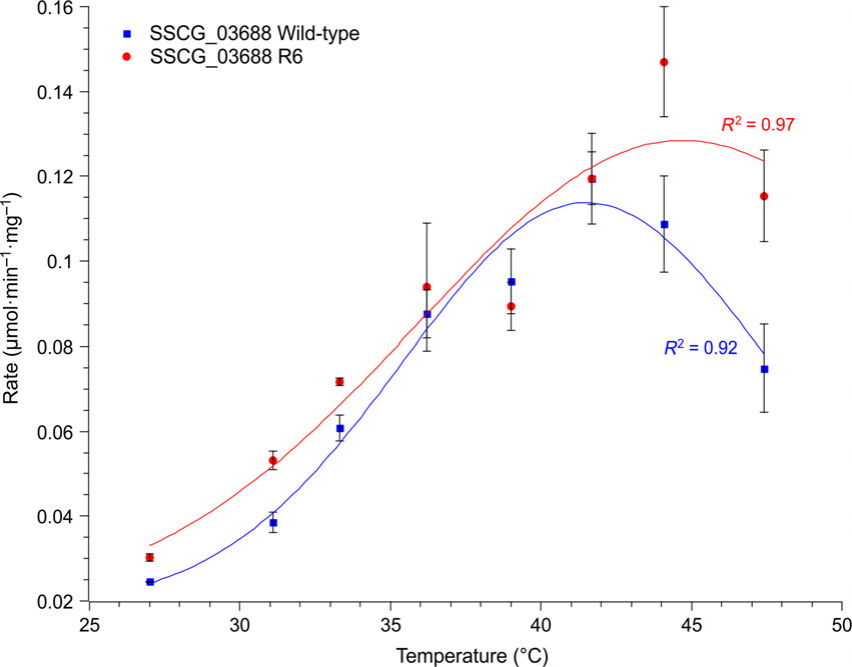
Thermoactivity profile of the two SSCG_03688 sesquiterpene synthases, SSCG_03688 wild‐type and the most truncated mutant SSCG_03688 R6. The error bars represent the standard error of mean for three replicates.

**Figure 6:**
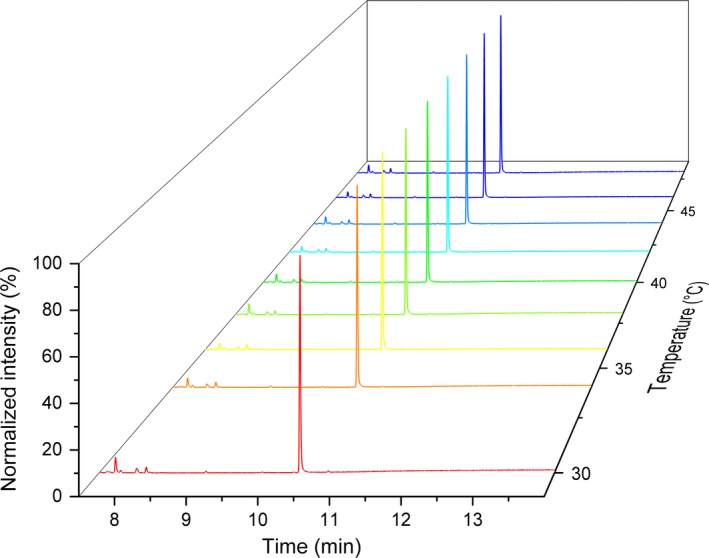
Normalized MS chromatograms showing the GC‐MS product profile of the SSCG_03688 R6 mutant at different temperatures. The major peak is τ‐muurolol.

The thermoactivity of the R6 truncated mutant was compared with that of the wild‐type between 27 and 47 °C using the malachite green assay 30. The new mutant had consistently higher activity at all temperatures. These results followed the same trend when the experiments were repeated using the malachite green assay protocol described by Vardakou *et al*. 30 (Fig. 5), which appeared to give reduced variability between replicates compared to the GC‐MS protocol. The malachite green assay protocol also gave consistently higher figures for activity compared to the GC‐MS protocol. However, GC‐MS data were required to determine the sesquiterpene product profile, which remained unchanged at each temperature for both the wild‐type and the mutants (Fig. 6).

## Discussion

*Roseiflexus* species are natural thermophiles that have been isolated from phototrophic mats in hot springs, growing at 60 °C 31. Genome sequences are available for *Roseiflexus* sp. RS‐1 and *R. castenholzii* DSM 13941, and both contain genes encoding TSs. Coincidentally, both enzymes have previously been characterized as τ‐muurolol synthases as part of a wider screen demonstrating that bacterial TSs are much more widespread than previously appreciated, but no investigation was made into their thermostability 16. In this work, RoseRS_3509 from *Roseiflexus* sp. RS‐1 and Rcas_0622 from *R. castenholzii* DSM 13941 have been characterized as naturally thermostable TSs and demonstrated to have activity *in vitro* up to 78 °C, which represents the highest temperature for which activity in this class of enzyme have been observed (including a TS computationally designed for improved thermostability) 18.

Bacterial TSs have a simpler structure than their plant counterparts, lacking any N‐terminal domains. However, they retain the typical α‐helical fold of the catalytic components of TSs, despite having low primary sequence identity. This simpler structure is attractive from the prospect of protein engineering but the sluggish kinetics of mesophilic bacterial TSs (*k*_cat_ typically between 10^−2^ and 10^−3^ s^−1^) restricts their application 32. The larger plant TSs typically have *k*_cat_ values between 10^−1^ and 10^−2^ s^−1^ 32. Due to their low turnover, TSs are rate limiting in industrial terpene‐producing organisms and they are typically artificially overexpressed relative to the precursor pathway 33. We report *k*_cat_ values in a bacterial τ‐muurolol synthase at 65 °C which are similar, if not superior, to most plant enzymes. The mesophile τ‐muurolol synthase SSCG_03688, which converts the same substrate into the same product as our thermostable examples, presumably by the same reaction cascade, has a reported *k*_cat_ value two orders of magnitude lower 17. This is interesting as, while increased temperature typically accelerates enzyme catalysis up to the stability limits of the enzyme, it does not follow that thermostable enzymes have higher catalytic rates than their less thermostable counterparts. Indeed, it has been shown that thermostability is typically achieved from greater structural rigidity, such that the appropriate mobility for good catalysis is only achieved at higher temperatures 34. The dramatic enhancement in *k*_cat_ going from an enzyme which works optimally at 40 °C to one that works optimally at > 60 °C, but with broadly similar tertiary structure suggests that the factors which provide thermostability do not significantly affect catalysis (i.e. the active site is not compromised by increased structural rigidity), so that providing a more thermostable framework does allow the benefits of temperature on accelerating catalysis to be realized in this class of enzymes. Given the evidence that the α‐helical fold structure of the catalytic component of class‐I TSs essentially provides a cage within which a carbocation can be generated and rearranged, it is possible to envisage that increased rigidity of the cage (providing thermostability) is independent of the mobility of catalytically functional residues. If this is true, then understanding how the α‐helical fold structure is stabilized could provide strategies for thermostabilization of all single domain class‐I TSs. Indeed, the DSC experiments demonstrated that the putative closed conformation of these TSs, induced by the presence of Mg^2+^ and pyrophosphate, resulted in an increase in *T*_m_ in all cases.

By modelling both the thermostable TSs and SSCG_03688 on a closely related structure, several differences could be observed, the most obvious being the extended C‐terminal tail of the mesophile enzyme. Previous work has shown that even a small truncation of a TS C terminus can result in an increase in thermostability 19. Sequentially truncating the C terminus of SSCG_03688 resulted in a moderate but consistent increase in thermostability, as determined by CD (Fig. 3B). The trend in *T*_m_ was also observed by DSC for SSCG_03688 and the R6 mutant, and the *T*_m_ values obtained for the open enzyme conformations (in the absence of Mg^2+^ and pyrophosphate) were comparable between the two techniques. Moreover, by truncating the C terminus of SSCG_03688, the difference between the *T*_m_ of the closed and open states was reduced, implying that the truncation leads to a reduction in flexibility around the active‐site lid without impacting negatively on the kinetics 29. Although we were not able to remove the C‐terminal tail entirely without affecting protein folding, we were able to truncate the tail by 57 amino acids and still obtain a functional and soluble enzyme, which additionally had a significantly higher *k*_cat_ value (Table 2). Referring to the model overlay, it can be observed that further truncation would remove a short helical region in the tail, which appears to be essential for soluble expression of the protein (Fig. 2). In experiments with the most truncated mutant, it was observed that there was an overall trend towards higher thermoactivity (Fig. 5), demonstrating that the C‐terminal tail in the wild‐type has a detrimental effect on thermostability.

While removing 57 amino acids only resulted in a moderate change in thermostability, it did not negatively affect either the kinetics or product profile of the enzyme. This is the second example (along with the directed evolution work from Lauchli *et. al*. 19) of C‐terminal truncation coinciding with improved thermostability of a TS, suggesting that a more minimal (truncated) enzyme is a reasonable starting place for thermostability improvement. It is interesting that such a substantial truncation does not result in a loss of catalytic efficiency, and implies that the unstructured C‐terminal tail has some other function, such as influencing protein localization through some kind of protein–protein interaction.

It is interesting that thermophilic bacteria have enzymes for making volatile terpenes. Both thermostable TS gene sequences are found in operons containing genes coding for a putative type 11 methyl transferase and an antisigma‐factor antagonist, and are flanked by a genes for a transmembrane protein and a beta‐lactamase. *Roseiflexus* species are typically found in the diverse communities of microbial mats in hot springs 31, so it is possible that a diffusible terpene might be used as a signalling molecule.

Given that these naturally thermostable TSs share a comparable level of sequence identity to over a thousand bacterial putative sesquiterpene synthases as they do to SSCG_03688, it is clear they can be used as a model for improvement of class‐I TS thermostability, and that further structural studies might provide fundamental insights into TS engineering.

## Experimental procedures

### Chemicals

The pure (E,E)‐FPP standard was purchased from Isoprenoids (Tampa, Florida, USA) and used for all TS assays reported. Gene sequences were optimized for codon usage in *E. coli* and purchased from GeneArt (ThermoFisher Scientific, Paisley, UK). DNA‐modifying enzymes were purchased from Life Technologies (Paisley, UK). Bradford protein assay dye reagent was obtained from Bio‐Rad (Hertfordshire, UK). All other chemicals were purchased from Sigma‐Aldrich except where indicated otherwise.

### Strains, plasmids and growth conditions

All cloning and plasmid propagation were carried out in *E. coli* BIOBlue chemically competent cells (Bioline, London, UK). All protein expression was carried using *E. coli* BL21(DE3). The three TS genes, *roseRS_3509, rcas_0622* and *sscg_03688*, were inserted between the *Nhe*I and *Eco*RI restriction sites of pET28a, for the expression of N‐terminal His_6_‐tagged proteins. *Escherichia coli* cultures were grown at 37 °C and 250 r.p.m. in Luria–Bertani (LB) medium supplemented with 50 μg·mL^−1^ of kanamycin for selection of the plasmid except when specified otherwise.

### Protein production and purification

For the production of RoseRS3509 and Rcas_0622, 1 mL of overnight *E. coli* BL21(DE3) culture transformed with the appropriate plasmid was used to inoculate 1 L of LB. Expression of genes controlled by the T7‐promoter was induced by adding 0.1 mm isopropyl β‐D‐1‐thiogalactopyranoside (IPTG) to cultures once they had reached an optical density (OD_600_) of 0.5. Cultures were harvested 20 h postinduction and the cells were pelleted by centrifugation (2100 ***g***, 4 °C, 10 min). The supernatant was discarded, and cell pellets were resuspended in Buffer A (20 mm imidazole, 20 mm Tris, 300 mm NaCl, pH 8), before being lysed by sonication (3 × 15 s pulses, samples kept on ice). Cell debris was separated by centrifugation (12 000 ***g***, 10 min), and the supernatant taken as the soluble protein fraction. The soluble fraction was loaded onto a column containing pre‐equilibrated TALON® metal affinity resin (Clontech Laboratories, Saint‐Germain‐en‐Laye, France) as per the manufacturer's instructions. The column was washed with 20 column volumes of Buffer A, and eluted with Buffer B (500 mm imidazole, 20 mm Tris, 300 mm NaCl, pH 8). Protein size and purity was checked using SDS/PAGE (both proteins expressed well under these conditions and resulted in a single major band at ~ 40 kDa with > 95% purity), and buffer was exchanged into TS buffer 1 [25 mm 2‐(*N*‐morpholino)ethanesulfonic acid (MES), 50 mm Tris, 25 mm *N*‐cyclohexyl‐3‐aminopropanesulfonic acid (CAPS), 100 mm NaCl, 10 mm MgCl_2_] using Amicon® Ultra 15 mL spin concentrators [10 kDa MWCO (molecular weight cut‐off), Watford, UK]. Protein was then used immediately for assays, or 10% glycerol was added and samples aliquoted into tubes for storage at −80 °C. Production of SSCG_03688 was adapted from the method used by Hu *et al*. 17. Briefly, the conditions were the same as above with the following exceptions; immediately post induction, cultures were grown at 18 °C for the 20‐h protein expression. For the lysis and purification, Buffer C [50 mm tris(hydroxymethyl)aminomethane hydrochloride salt (Tris/HCl), 0.5 m NaCl, 20 mm imidazole, 5 mm 2‐mercaptoethanol, pH 7.5] was used in place of Buffer A, and Buffer D (50 mm Tris/HCl, 0.5 m NaCl, 500 mm imidazole, 5 mm 2‐mercaptoethanol, pH 7.5) was used in place of Buffer B for elution. Protein size and purity was checked using SDS/PAGE (the wild‐type and all mutant bands had > 95% purity; the wild‐type band was at ~ 48 kDa and truncated mutant proteins were visibly and sequentially smaller on the gel). Eluted SSCG_03688 protein was exchanged into TS buffer 2 [TS2; 50 mm piperazine‐*N*,*N*′‐bis(2‐ethanesulfonic acid) (PIPES), 10 mm MgCl_2_, 100 mm NaCl, 5 mm 2‐mercaptoethanol, pH 7.5]. Finally, aliquots were stored at −80 °C in 20% glycerol. All protein stocks were quantified using the Bradford protein assay.

### Terpene synthase activity assay

Assays were adapted from the GC vial assay described by Garrett *et al*. 25. Stock enzyme solution was diluted to 50 μm concentration by addition of TS buffer. Final 200 μL reaction volumes were prepared in 500 μL tubes by addition of 4 μL of 50 μm enzyme solution to 196 μL TS buffer, and the reaction was started by adding the appropriate amount of FPP stock (1 mg·mL^−1^, 7 : 3 methanol : 10 mm NH_4_OH). For the GC vial assays, reactions were stopped by snap freezing in liquid nitrogen, and 150 μL of hexane spiked with 10 μg·mL^−1^ caryophyllene internal standard was added to the frozen sample. Tubes were then vortexed until the reaction solution was completely melted (~ 1 min), and then centrifuged (2100 ***g***, 2 min) and the organic layer transferred to a 200 μL vial insert for analysis by GC‐MS. For assays carried out at higher temperatures, tubes containing buffer were incubated in a thermal cycler block at the desired temperature for at least 10 min prior to the start of the reaction. All TS assays were carried out in triplicate, and errors reported as standard error of the mean.

### Thermoactivity of RoseRS3509 and Rcas_0622

Assays were carried out with an enzyme concentration of 1 μm over 1 min at a range of temperatures 50–84 °C, with a substrate concentration of 58 μm. Reaction buffer was prewarmed at each temperature in a thermocycler for at least 10 min, the enzyme was added, and after a 15‐s equilibration time, the reaction was started by the addition of FPP. The samples were then processed as before using the GC vial assay protocol. The data were fit to a simple Gaussian function to aid visualization (Fig. 1A). The errors are reported as standard error of the mean.

### Thermoactivity of SSCG_03688 variants using the malachite green assay

Thermoactivity of SSCG_03688 wild‐type and mutant were both determined using the recently developed malachite green protocol for TS activity in order to confirm the trends observed using the GC‐MS protocol 30. Assays were carried out with an enzyme concentration of 1 μm over 8 min at a range of temperatures 30–47 °C, with a substrate concentration of 58 μm. Reaction buffer was prewarmed at each temperature in a thermocycler for at least 10 min, the enzyme was added, and after a 15‐s equilibration time, the reaction was started by the addition of FPP. The data were fit to a simple Gaussian function to aid visualization (Fig. 5). Assays were carried out in triplicate, and the errors were reported as standard error of the mean.

### GC‐MS and GC‐FID analysis

Analysis of terpene products was carried out on an Agilent 7890B GC coupled to a 5977A mass spectrometer (Agilent Technologies, Stockport, UK), and separation was achieved using a DB‐FFAP capillary GC column (30 m × 250 μm× 0.25 μm). Of sample, 1 μL was injected into the column, which was held at 40 °C for 1 min. The temperature was then ramped 20 °C per min to a final temperature of 250 °C, which was held for a further 8 min. Terpene compounds typically eluted between 5 min and 13 min, and the total method time was 19.5 min, and were monitored on both the MS and FID detector. The product peak area from the FID chromatogram was divided by the internal standard peak area, then multiplied by the caryophyllene standard concentration to generate a ‘caryophyllene equivalents’ value for product concentration in the organic extract. Due to the unit carbon response of the FID detector, and in the absence of a commercially available τ‐muurolol standard, caryophyllene is suitable for the relative quantification of sesquiterpene products 35.

### Steady‐state kinetics

Assays were carried out with an enzyme concentration of 1 μm and substrate concentrations of 1.2, 2.3, 4.6, 12, 23, 46 and 92 μm using the GC‐FID vial assay protocol described above. For the thermostable TSs, reactions were carried out in triplicate at 65 °C and were stopped after 1 min. For SSCG_03688 and the truncated mutant variant, assays were carried out in triplicate at 30 °C and were stopped after 5 min. The data were fit to the Michaelis–Menten equation and kinetic values were derived using sigmaplot Version 12.3 (Systat Software, San Jose, CA, USA).

### Circular dichroism (CD) protein melts

Fresh enzyme was purified and the elution buffer exchanged with a buffer compatible with the CD instrument (100 mm NaF, 10 mm phosphate buffer, pH 8.0). Buffer exchange was carried out by dialysis (10 kDa MWCO) overnight, and 250 μL of sample (enzyme concentration 5 μm) was pipetted into a 1 mm path length quartz cuvette. CD was monitored on a Chirascan CD Spectrometer (Applied Photophysics Ltd, Surrey, UK) at 222 nm for 5 s at 1 °C intervals between 10 and 95 °C.

*T*_m_ determination – The data were fit using Eqn (1) for a simple two‐state transition,

(1) $$\theta_{222\;\mathrm{nm}}=\frac{b_{u}+m_{u}T+\left(b_{f}+m_{f}T\right)K_{u}}{1+K_{u}}$$

where

(2) $$K_{u}=\text{exp}\left(\frac{\Delta H(1-\frac{T}{T_{m}})}{RT}\right)$$

where *b* and *m* are the slope and intercept of the unfolded (*u*) and folded (*f*) baseline respectively; *T*_m_ is the melting temperature of the protein; and Δ*H* is the van′t Hoff enthalpy of unfolding at *T*_m_. Data fitting was carried out using scidavis data analysis software (http://scidavis.sourceforge.net/about.html).

### Differential scanning calorimetry

Analysis of protein by DSC was conducted on a μSC (Setaram Instrumentation, Caluire, France). It was determined that > 800 μg of protein was required in order to observe a consistent signal for protein melting experiments. Sufficient stock protein was diluted to 500 μL (final volume) using either TS buffer 2 (25 mm MES, 50 mm Tris, 25 mm CAPS, 100 mm NaCl) or TS buffer 3 (TS buffer 2 with 10 mm MgCl_2_ and 1 mm Na_4_P_2_O_7_). The buffers were pH 8.0 for RoseRS_3509 and Rcas_0622 and pH 7.5 for the SSCG_03688 variants. The 500 μL protein samples were sealed inside a measurement cell, and the heat flow was determined against a reference cell, which contained an equivalent volume of the appropriate buffer. The instrument was programmed to hold the samples at 20 °C for 5 min, before it increased the temperature by 1 °C per min up to 95 °C (75 °C for SSCG_03688 experiments). Data were baseline corrected using the Calisto Processing software (v 1.41, Setaram Instrumentation).

### Sequence alignment and modelling

The protein sequences for the two thermostable τ‐muurolol synthases (RoseRS_3509 Accession Number: WP_011958209.1, Rcas_0622 Accession Number: WP_012119179.1) were aligned with that of SSCG_03688 (Accession Number EDY50541.1) using the T‐Coffee web server alignment tool 36, 37. The sequences of RoseRS_3509 and SSCG_03688 were both initially modelled against the template crystal structure of selinadiene synthase (PDB entry http://www.rcsb.org/pdb/search/structidSearch.do?structureId=4OKM.4.A) using the SWISS‐MODEL web server 38. The sequences were then modelled using iterative threading assembly refinement (I‐TASSER server) 27, 28. The resultant.pdb files from the I‐TASSER modelling were visualized (Fig. 2) using pymol (The pymol Molecular Graphics System, Version 1.8 Schrödinger, LLC, Cambridge, UK).

### Preparation of tris(tetra‐*n*‐butylammonium) hydrogen phosphate

A solution of disodium dihydrogen pyrophosphate (3.33 g, 15 mmol) in 15 mL of 10% (v/v) aqueous ammonium hydroxide was passed through a column (3 × 9 cm) of Dowex 50WX8 (100–200 mesh) cation exchange resin and subsequently flushed with deionized water (110 mL). The resulting solution (pH = 1) was immediately titrated to a pH of 7.3 with 40% (w/w) aqueous tetra‐*n*‐butylammonium hydroxide followed by lyophilization of the entire reaction mixture to leave a hygroscopic white solid (12.7 g, 96%). All analytical data match that of the original report 39. This crude reaction material was used in the following steps with no further purification.

### Preparation of farnesyl chloride

In a flame dried, two necked 250 mL round‐bottomed flask under a blanket of nitrogen was charged *N*‐chlorosuccinimide (292 mg, 2.2 mmol) dissolved in anhydrous dichloromethane (DCM, 100 mL). The reaction was cooled to −30 °C followed by the dropwise addition of dimethyl sulphide (149 mg, 2.4 mmol) under vigorous stirring of the heterogeneous mixture. The reaction was warmed to 0 °C for 5 min before cooling back down to −30 °C. Farnesol (444 mg, 2 mmol) was dissolved in anhydrous DCM (10 mL) and was added dropwise to the milky white suspension. The reaction was slowly warmed to 0 °C over 1 h and stirred at this temperature for a further 1 h. After warming to room temperature and stirring for an additional 15 min, the now clear solution was extracted with a cold saturated brine solution (50 mL) with further extraction of the aqueous layer (DCM, 2 × 50 mL). The combined organic extracts were further washed with a saturated brine solution (50 mL) and dried over magnesium sulphate before the removal of the solvent *in vacuo*. The crude colourless oil (450 mg, 94%) matched the analytical data originally reported 39 and was used without further purification.

### Preparation of FPP tetra‐*n*‐butylammonium salt

In a flame dried, two necked 25 mL round‐bottomed flask, under a blanket of nitrogen was added a solution of the crude tris(tetra‐*n*‐butylammonium) hydrogen pyrophosphate (1.8 g, Eqn (2)) dissolved in anhydrous acetonitrile (2 mL). To this was added a solution of the crude farnesyl chloride (240 mg, Eqn (1)) in anhydrous acetonitrile (2 mL) and the reaction was stirred at room temperature for 2 h. The solvent was then removed *in vacuo* to yield the crude material as an orange oil (2.2 g).

The oil was dissolved in methanol to a stock concentration of 0.5 g·mL^−1^, and stored at −20 °C. The stock was diluted in methanol : 10 mm aqueous NH_4_OH (7 : 3) in preparation for assays. FPP content was determined by comparison of activity compared to a pure standard, and was typically 15–20% without any further work‐up steps.

## Author contributions

MQS designed and conducted most of the experiments, analysed the results and wrote the paper. EAN conducted the experiments on the SSCG_03688 enzyme and its mutants. SM optimized the expression of Rcas_0622. MH synthesized the FPP tetra‐*n*‐butylammonium salt. DJL conceived the idea for the project and helped to write the paper.

## Conflict of interest

The authors declare they have no conflicts of interest with the contents of this article.
